# Supplementary material for: The Role of Risk or Contributory Death Factors in Methadone-Related Fatalities: A Review and Pooled Analysis
Source: Metabolites. 2021 Mar 22;11(3):189. doi: 10.3390/metabo11030189 (PMC8004630; doi:10.3390/metabo11030189)
Supplement: Supplementary file 1 [file metabolites-11-00189-s001.pdf]

Figure S1. Prisma flow-chart of papers and cases selection from the literature and from the in house casuistry.

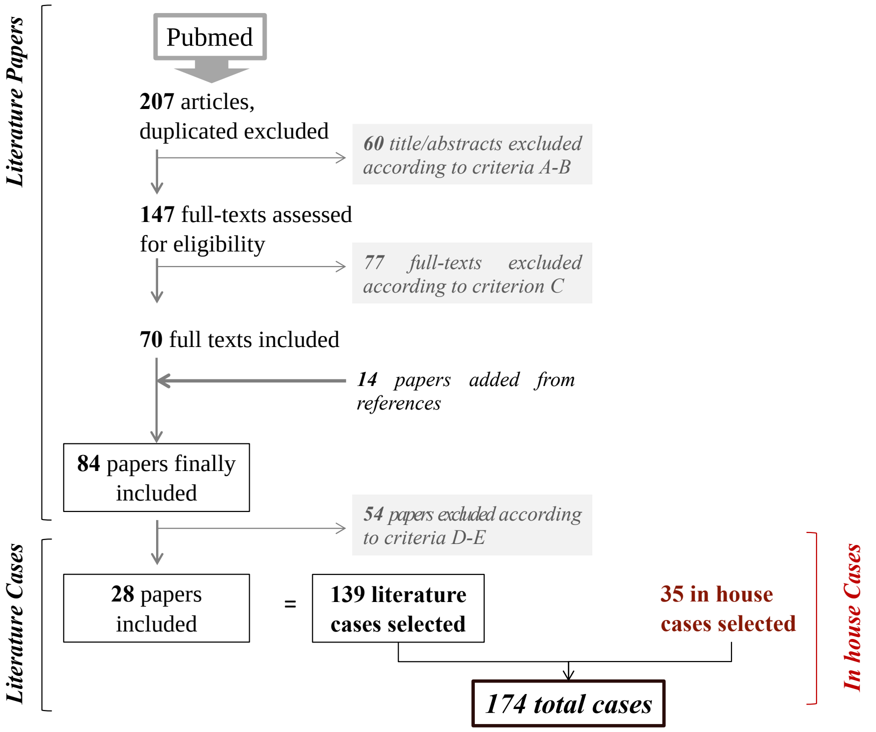

Table S1. Literature review – Included papers

Ref..	Authors	Title	Year	Type of article	Journal
[90]	Akhgari et al.	Forensic toxicology perspectives of methadone-associated deaths in Tehran, Iran, a 7-year Overview	2018	Retrospective case series	Basic & Clinical Pharmacology & Toxicology
[39]	Albion et al.	Contributing factors to methadone-related deaths in Ontario	2010	Retrospective case series	The American journal of forensic medicine and pathology
[40]	Andersson et al.	Investigating opioid-related fatalities in Southern Sweden: contact with care-providing authorities and comparison of substances	2020	Retrospective case series	Harm Reduction Journal
[41]	Argo et al.	A possible biomarker for methadone related deaths	2017	Original article	Journal of Forensic and Legal Medicine
[42]	Aromatario et al.	Methadone related deaths: identifying the vulnerable patients	2013	Case report	Journal of Forensic Toxicology and Pharmacol
[86]	Ballesteros et al.	Increase in Deaths Due to Methadone in North Carolina	2003	Retrospective case series	JAMA
[16]	Bell et al.	Comparing overdose mortality associated with methadone and buprenorphine treatment	2009	Retrospective case series	Drug and Alcohol Dependence
[18]	Bernard et al.	Methadone-related deaths in Norway	2013	Retrospective case series	Forensic Science International
[109]	Bernard et al.	Characteristics of methadone-related fatalities in Norway	2015	Retrospective case series	Journal of Forensic and Legal medicine
[43]	Bonsignore et al.	Fatal methadone intoxication in an infant listed as a homicide	2016	Case report	International Journal of Legal Medicine
[78]	Bryant et al.	Overdose deaths attributed to methadone and heroin in New York City, 1990–1998	2003	Retrospective case series	Addiction
[110]	Buchard et al.	Postmortem blood concentrations of R- and S-enantiomers of methadone and EDDP in drug users: influence of co-medication and p-glycoprotein genotype.	2010	Original article	Journal of Forensic Sciences
[44]	Buster et al.	An increase in overdose mortality during the first 2 weeks after entering or re-entering methadone treatment in Amsterdam	2002	Retrospective case series	Addiction
[101]	Byard et al.	Diabetic ketoacidosis – a possible complicating factor in deaths associated with drug overdose		Case report	Medicine, Science and the Law
[45]	Caplehorn et al.	Fatal methadone toxicity: signs and circumstances, and the role of benzodiazepines	2002	Retrospective case series	Australian and New Zeland journal of public health
[74]	Caplehorn et al.	Methadone dose and post-mortem blood concentration	2002	Retrospective case series	Drug and Alcohol Review

[98]	Carson et al.	Identification at autopsy of pulverized pills in lungs of a first-time methadone user	2009	Case report	Journal of Forensic and Legal Medicine
[32]	Chugh et al.	A community-based evaluation of sudden death associated with therapeutic levels of methadone	2008	Prospective case series	Am J Med.
[23]	Corkery et al.	The effects of methadone and its role in fatalities	2004	Review	Human Psychopharmacology
[46]	Couper et al.	Fatal methadone intoxication in an infant	2005	Case report	Forensic Science International
[89]	Dai et al.	Quantifying enhanced risk from alcohol and other factors in polysubstance-related deaths	2020	Retrospective case series	Forensic Science International
[47]	Darke et al.	The comparative toxicology and major organ pathology of fatal methadone and heroin toxicity cases	2010	Retrospective case series	Drug and Alcohol Dependence
[102]	Drummer etal.	Recent Trends in Narcotic Deaths	2005	Review	Therapeutic Drug Monitoring
[97]	Eiden et al.	Methadone-related deaths in Montpellier and Region, from 2000 to 2010	2012	Retrospective case series	Therapie
[81]	Ernst et al.	Methadone-related deaths in Western Australia 1993-99	2002	Retrospective case series	Australian and New Zealand Journal of Public Health
[106]	Fahey et al.	Sudden death in an adult taking methadone: lessons for general practice	2003	Case report	British Journal of General Practice
[77]	Fiddler et al.	A review of GP records relating to methadone-associated deaths in the Lothian region of Scotland 1997-9.	2001	Retrospective case series	Journal of Substance Use
[48]	Fields et al.	Parent and metabolite opioid drug concentrations in unintentional deaths involving opioid and benzodiazepine combinations	2015	Retrospective case series	Journal of forensic sciences
[80]	Fugelstad et al.	Methadone maintenance treatment: the balance<br>between life-saving treatment and fatal poisonings	2007	Retrospective case series	Addiction
[87]	Fugelstad et al.	Methadone-related deaths among youth and young adults in Sweden 2006-15	2020	Retrospective case series	Addiction
[38]	Gagajewski et al.	Methadone-related deaths in Hennepin County, Minnesota: 1992-2002	2003	Retrospective case series	Journal of Forensic Sciences
[49]	Gao et al.	Risk-factors for methadone-specific deaths in Scotland's methadone-prescription clients between 2009 and 2013	2016	Retrospective case series	Drug and Alcohol Dependence
[91]	Gharehdaghi	Suspected methadone toxicity: from hospital to autopsy bed	2013	Retrospective case series	Basic & Clinical Pharmacology & Toxicology

[83]	Graham et al.	Methadone- and heroin-related deaths in Florida	2008	Retrospective case series	The American Journal of Drug and Alcohol Abuse
[108]	Grass et al.	Methadone and its role in drug-related fatalities in Cologne 1989–2000	2003	Retrospective case series	Forensic Science International
[84]	Green et al.	Methadone maintenance programs-a two-edged sword?	2001	Retrospective case series	American Journal of Forensic Medicine & Pathology
[50]	Häkkinen et al.	Comparison of fatal poisonings by prescription opioids	2012	Retrospective case series	Forensic Science International
[95]	Häkkinen et al.	Prescription opioid abuse based on representative postmortem toxicology	2014	Retrospective case series	Forensic Science International
[72]	Heinemann et al.	Methadone-related fatalities in Hamburg 1990–1999: implications for quality standards in maintenance treatment?	2000	Retrospective case series	Forensic Science International
[51]	Holm et al.	Distribution of enantiomers of methadone and its main metabolite EDDP in human tissues and blood of postmortem cases	2015	Original article	Journal of forensic sciences
[34]	Iwersen-Bergmann et al.	Intravenous methadone application as a serious risk factor for an overdose death: methadone-related fatalities in Hamburg from 2007 to 2012	2014	Retrospective case series	International Journal of Legal Medicine
[79]	Jones et al.	Blood methadone concentrations in living and deceased persons: variations over time, subject demographics, and relevance of coingested drugs	2012	Retrospective case series	Journal of Analytical Toxicology
[104]	Karlovsek	Illegal drugs-related fatalities in Slovenia	2004	Retrospective case series	Forensic Science International
[52]	Kintz et al.	Methadone as a chemical weapon: two fatal cases involving babies	2005	Case report	Therapeutic Drug Monitoring
[53]	Laberke et al.	Trends in methadone-related deaths in Zurich	2010	Retrospective case series	International Journal of Legal Medicine
[93]	Lev et al.	Methadone related deaths compared to all prescription related deaths	2015	Retrospective case series	Forensic Science International
[54]	Li et al.	Fatal methadone poisoning in children: Maryland 1992-1996	2009	Retrospective case series	Substance Use & Misuse
[92]	Lusetti et al.	Therapeutic and recreational methadone cardiotoxicity	2016	Retrospective case series	Journal of Forensic and Legal Medicine
[55]	Madden et al.	The methadone epidemic: methadone-related deaths on the rise in Vermont	2011	Retrospective case series	American journal of forensic medicine and pathology
[56]	Mijatović et al.	Methadone-related deaths – epidemiological, pathohistological, and toxicological traits in 10-year retrospective study in Vojvodina, Serbia.	2014	Retrospective case series	Journal of forensic sciences
[107]	Mikolaenko et al.	A review of methadone deaths in Jefferson County, Alabama	2002	Retrospective case series	American journal of forensic medicine and pathology

[33]	Milroy et al.	Methadone deaths: a toxicological analysis	2000	Retrospective case series	Journal of clinical pathology
[57]	Mistry et al.	Methadone toxicity in infants: a report of two fatalities	2010	Case report	Forensic Science Medicine and Pathology
[36]	Musshoff et al.	Methadone substitution: medicolegal problems in Germany	2003	Retrospective case series	Forensic Science International
[28]	Nielsen et al.	Evaluation of poly-drug use in methadone-related fatalities using segmental hair analysis	2015	Retrospective case series	Forensic Science International
[58]	Nielsen et al.	Evaluation of metabolite/drug ratios in blood and urine as a tool for confirmation of a reduced tolerance in methadone-related deaths in Denmark	2013	Retrospective case series	Drug and Alcohol Dependence
[59]	Okic et al.	Opioid overdose mortality in Kansas, 2001-2011: toxicologic evaluation of intent	2013	Retrospective case series	Journal of analytical toxicology
[60]	Palmiere et al.	An unusual case of accidental poisoning: fatal methadone inhalation	2011	Case report	Journal of Forensic Sciences
[61]	Palmiere et al.	Parental substance abuse and accidental death in children	2010	Case report	Journal of Forensic Sciences
[62]	Paulozzi et al.	A comparison of drug overdose deaths involving methadone and other opioid analgesics in West Virginia	2009	Retrospective case series	Addiction
[63]	Perret et al.	Lethal methadone intoxications in Geneva, Switzerland, from 1994 to 1998	2000	Retrospective case series	Addiction
[94]	Petrushevska et al.	Drug-related deaths between 2002 and 2013 with accent to methadone and benzodiazepines	2015	Retrospective case series	Journal of Forensic and Legal Medicine
[35]	Pilgrim et al.	A review of methadone deaths between 2001 and 2005 in Victoria, Australia	2013	Retrospective case series	Forensic Science International
[105]	Pirnay et al.	A critical review of the causes of death among post-mortem toxicological investigations: analysis of 34 buprenorphine-associated and 35 methadone-associated deaths	2004	Retrospective case series	Addiction .
[76]	Seymour et al.	The role of methadone in drug-related deaths in the west of Scotland	2003	Retrospective case series	Addiction .
[64]	Shields et al.	Methadone toxicity fatalities: a review of medical examiner cases in a large metropolitan area	2007	Retrospective case series	Journal of forensic sciences
[65]	Simonsen et al.	Fatal poisoning among patients with drug addiction	2015	Retrospective case series	Danish medical journal
[66]	Simonsen et al.	Fatal poisoning in drug addicts in the Nordic Countries in 2012	2015	Retrospective case series	Forensic Science International
[67]	Sorg et al.	Additive effects of cointoxicants in single-opioid induced deaths	2016	Retrospective case series	Academic Forensic Pathology
[100]	Soyka et al.	Fatal poisoning in methadone and buprenorphine treated patients -- are there differences?	2006	Retrospective case series	Pharmacopsychiatry

[103]	Steentoft et al.	Fatal poisoning in Nordic drug addicts in 2002	2006	Retrospective case series	Forensic Science International
[68]	Sutlovic et al.	Methadone maintenance treatment: a 15-year retrospective study in split-Dalmatia County, Croatia	2018	Retrospective case series	Therapeutic Drug Monitoring
[19]	Tjagvad et al.	Methadone-related overdose deaths in a liberal opioid maintenance treatment programme	2016	Retrospective case series	European Addiction Research
[96]	Tormey et al.	Methadone, codeine and acute haemorrhagic necrotising pancreatitis: which came first?	2013	Case report	Forensic Science International
[75]	Valmana et al.	Methadone-related deaths: data from 18 coroners' jurisdictions in England	2000	Retrospective case series	Medicine Science and the Law
[88]	Van Den Broecke et al.	Methadone-related fatalities: review in the Ghent district between 1978-2008	2012	Retrospective case series	Acta Clinica Belgica
[99]	Verhoek-Oftedahl et al.	Drug intoxication deaths involving methadone, 2004-2005	2007	Retrospective case series	Medicine and health, Rhode Island
[69]	Vignali et al.	Methadone-related deaths. A ten year overview	2015	Retrospective case series	Forensic Science International
[73]	Vormfelde et al.	Death attributed to methadone	2001	Review	Pharmacopsychiatry
[85]	Wagner-Servais	Methadone-related deaths associated with faulty induction procedures	2003	Retrospective case series	Journal of Maintenance in the Addictions
[70]	Wikner et al.	Opioid-related mortality and filled prescriptions for buprenorphine and methadone	2014	Retrospective case series	Drug and alcohol review
[71]	Wolf et al.	Methadone-related deaths in Palm Beach County	2004	Retrospective case series	Journal of Forensic Sciences
[37]	Wolff	Characterization of methadone overdose: clinical considerations and the scientific evidence	2002	Review	Therapeutic Drug Monitoring
[82]	Wunsch et al.	Opioid deaths in Rural Virginia: a description of the high prevalence of accidental fatalities involving prescribed medications	2009	Retrospective case series	American Journal on Addictions

Table S2. Literature cases, with extracted data and classification.

Ref: references

Gender. F: female, M: male.

Post-mortem examination. H: heart; L: lung.

neg: not detected in blood; np: data not provided.

6-MAM: 6-monoacetylmorphine; BEG: benzoylecgonine; BZDs: benzodiazepine

Other drugs. THC: tetrahydrocannabinol; THC-COOH: tetrahydrocannabinolic acid.

D: drugs; N: naïve/non-tolerant; P: pulmonary; C: cardiac.

CN: controls. CA: cases

Ref.	Age (yo)	Gender	Naive/non tolerant	Post-mortem examination	Methadone (ng/mL)	6-MAM	Morphine	Codeine	Cocaine	BEG	BZDs	Other drugs	Disease or condition	Group
[41]	37	M	No	H: interstitial fibrosis, concentric left ventricular hypertrophy, critical three-vessel coronary artery stenosis<br>L: hemorrhagic edema, chronic lymphocytic bronchitis	270	neg	neg	neg	neg	neg	neg	neg	P, C	CA
	42	M	No	H: enlargement, myocyte hypertrophy; endocardial fibrosis. fibro-lipomatous infiltration<br>L: hemorrhagic edema, acute bronchopneumonia	610	neg	neg	neg	neg	neg	neg	neg	C	CA
	20	M	No	H: enlargement, myocyte hypertrophy, contraction band necrosis<br>L: hemorrhagic edema	370	neg	neg	neg	neg	neg	neg	neg	C	CA
	35	M	No	H: left ventricular chamber dilatation, pace-maker<br>L: edema, emphysema, granulomatous inflammation	890	neg	neg	neg	neg	neg	neg	neg	C	CA
	35	M	No	H: concentric hypertrophy of the left ventricle, signs of myocardial suffering<br>L: chronic bronchitis, alveolar edema	720	neg	neg	neg	neg	neg	neg	neg	P, C	CA

	25	F	No	L: hemorrhagic edema	900	neg	neg	neg	neg	neg	neg	neg	-	CN
	39	M	No	H: myocyte hypertrophy associated with mild perivascular fibrosis<br>L: hemorrhagic edema, panlobular emphysema with fibrotic thickening of the septa	580	neg	neg	neg	neg	neg	neg	neg	P	CA
	19	F	No	H: myocytolysis, contraction band necrosis, lipomatous infiltration of the right ventricle<br>L: hemorrhagic edema	270	neg	neg	neg	neg	neg	neg	neg	C	CA
	25	F	No	L: hemorrhagic edema	380	neg	neg	neg	neg	neg	neg	neg	-	CN
	25	M	No	H: myocyte hypertrophy<br>L: hemorrhagic edema, chronic lymphocytic bronchitis	530	neg	neg	neg	neg	neg	neg	neg	P	CA
[43]	2	M	No	H: aortic coartation and left ventricular hypertrophy<br>L: congestion	633	neg	neg	neg	neg	neg	neg	neg	C	CA
[54]	4	M	Yes	L: edema, congestion	600	neg	neg	neg	neg	neg	neg	neg	N	CA
	5	F	Yes	L: edema	600	neg	neg	neg	neg	neg	neg	neg	N	CA
	3	M	Yes	NP	400	neg	neg	neg	neg	neg	neg	neg	N	CA
[69]	39	M	Yes	NP	470	neg	neg	neg	neg	neg	Delorazepam	neg	N, D	CA
	27	M	Yes	NP	210	neg	neg	neg	neg	neg	Diazepam<br>Nordiazepam	neg	N, D	CA
	39	F	Yes	NP	1380	neg	neg	neg	neg	neg	7-aminoclonazepam<br>Lorazepam	Venlafaxine	N, D	CA
	25	M	Yes	NP	1080	neg	neg	neg	neg	neg		Citalopram	N, D	CA
	22	M	No	NP	520	neg	neg	neg	pos	pos	neg	neg	D	CA
	40	F	No	NP	490	neg	neg	neg	neg	neg	neg	Ethanol<br>Venlafaxine<br>Quetiapine	D	CA
	2	F	Yes	NP	200	neg	neg	neg	neg	neg	neg	neg	N	CA
	33	M	No	NP	210	neg	neg	neg	neg	neg	neg	Etha	D	CA
	32	M	No	NP	620	neg	neg	neg	neg	neg	neg	neg	-	CN
	28	M	No	NP	390	neg	neg	neg	neg	neg	neg	Ethanol	D	CA
	38	M	No	NP	3150	neg	neg	neg	neg	neg	neg	neg	-	CN
	29	M	No	NP	1460	neg	neg	neg	neg	neg	neg	neg	-	CN

	16	F	No	NP	690	neg	neg	neg	neg	pos	neg	neg	D	CA
	41	M	No	NP	640	neg	neg	neg	neg	neg	neg	Mirtazapine	D	CA
	26	M	No	NP	520	neg	neg	neg	neg	pos	neg	neg	D	CA
	2	M	No	NP	630	neg	neg	neg	neg	neg	neg	neg	-	CN
	39	F	No	NP	980	neg	neg	neg	neg	neg	Lorazepam	neg	D	CA
	39	M	No	NP	340	neg	neg	neg	neg	neg	neg	neg	-	CN
	36	M	No	NP	480	neg	neg	neg	neg	neg	neg	Ethanol	D	CA
	43	M	No	NP	1300	neg	neg	neg	neg	neg	neg	Ethanol Citalopram Levomeprom azine	D	CA
	24	M	No	NP	500	neg	neg	neg	neg	pos	neg	Ethanol	D	CA
	54	F	No	NP	980	neg	neg	neg	neg	neg	neg	neg	-	CN
	49	F	No	NP	3370	neg	neg	neg	neg	neg	Desalkylflurazepam	Ethanol	D	CA
	42	M	No	NP	1060	neg	neg	neg	neg	neg	neg	Ethanol Promazine	D	CA
	33	M	No	NP	970	neg	neg	neg	neg	neg	neg	neg	-	CN
	34	M	No	NP	920	neg	neg	neg	pos	pos	neg	Ethanol Citalopram	D	CA
	26	M	No	NP	590	neg	pos	neg	pos	pos	neg	Ethanol	D	CA
	30	M	No	NP	630	neg	neg	neg	neg	neg	neg	Ethanol Venlafaxine	D	CA
	42	M	No	NP	380	neg	neg	neg	neg	neg	Nordiazepam Delorazepam	Prometazine Levomeprom azine	D	CA
	29	M	No	NP	130	neg	neg	neg	neg	pos	neg	Ethanol	D	CA
	40	F	No	NP	1090	neg	neg	neg	neg	neg	neg	neg	-	CN
	44	M	No	NP	1190	neg	neg	neg	neg	neg	neg	Citalopram	D	CA
	43	M	No	NP	1500	neg	neg	neg	neg	pos	neg	neg	D	CA
	45	M	No	NP	2050	neg	neg	neg	neg	neg	neg	Sertraline	D	CA
	45	F	No	NP	1270	neg	neg	neg	neg	neg	Diazepam Nordazepam	neg	D	CA
	33	M	No	NP	1380	neg	neg	neg	neg	pos	neg	Levomeprom azine	D	CA

[60]	40	M	Yes	L: edema, congestion, alveolar and interstitial inflammation, desquamation of the ciliated respiratory epithelium	290	neg	neg	neg	neg	neg	neg	neg	N, P	CA
[61]	1	F	Yes	L: edema, congestion	1200	neg	neg	neg	neg	neg	neg	neg	N	CA
[34]	26	M	Yes	NP	260	neg	neg	neg	neg	neg	Diazepam Nordiazepam	Ethanol	N, D	CA
	34	M	No	NP	350	neg	pos	pos	neg	neg	neg	neg	D	CA
	17	F	No	NP	350	neg	pos	pos	pos	pos	neg	neg	D	CA
	26	M	No	NP	400	neg	pos	neg	neg	neg	Diazepam Nordiazepam Oxazepam	neg	D	CA
	42	M	No	NP	580	neg	pos	neg	neg	neg	Diazepam Nordiazepam Oxazepam	neg	D	CA
	40	F	No	NP	300	neg	pos	pos	neg	neg	Diazepam nordiazepam oxazepam lorazepam	Trimipramine Promethazine	D	CA
	60	M	No	NP	210	neg	neg	neg	neg	neg	Diazepam Nordiazepam Oxazepam	Chlorprothixene	D	CA
	24	F	No	NP	100	neg	neg	neg	neg	neg	neg	MDMA Amphetamines	D	CA
	37	M	No	NP	2500	neg	neg	neg	neg	neg	Diazepam Nordiazepam	Tramadol Venlafaxine	D	CA
	29	F	Yes	NP	370	neg	neg	neg	neg	neg	neg	Buprenorphine Doxepin Melperone Quetiapine	N, D	CA
	51	M	Yes	NP	410	neg	pos	pos	neg	neg	Diazepam Nordiazepam	neg	N, D	CA
	43	M	No	NP	1400	neg	neg	neg	neg	neg	neg	neg	-	CN
	38	F	No	NP	220	neg	pos	neg	pos	pos	neg	Levopromazine	D	CA
	33	M	No	NP	3130	neg	neg	neg	neg	neg	Diazepam Nordiazepam	neg	D	CA
	19	F	No	NP	740	neg	neg	neg	neg	pos	neg	THC	D	CA

												THC-COOH		
	23	M	Yes	NP	310	neg	neg	neg	neg	neg	neg	Ethanol<br>THC<br>THC-COOH	N, D	CA
	24	F	Yes	NP	570	neg	neg	neg	neg	neg	neg	neg	N	CA
	49	M	No	NP	560	neg	neg	neg	neg	neg	neg	neg	-	CN
	40	M	No	NP	670	neg	neg	neg	neg	pos	neg	neg	D	CA
	48	M	Yes	NP	330	neg	neg	neg	neg	neg	Diazepam	Doxepin	N, D	CA
	36	M	No	NP	310	neg	neg	neg	neg	neg	neg	neg	-	CN
	25	M	Yes	NP	390	neg	neg	neg	neg	neg	Diazepam<br>Nordiazepam	neg	D	CA
	29	M	No	NP	380	neg	neg	neg	neg	neg	neg	neg	-	CN
	19	M	No	NP	530	neg	neg	neg	neg	neg	Diazepam<br>Nordiazepam	Mirtazapine	D	CA
	22	F	No	NP	790	neg	neg	neg	neg	pos	neg	neg	D	CA
	40	M	No	NP	550	neg	neg	neg	neg	neg	Diazepam<br>Nordiazepam	THC-COOH	D	CA
	46	M	No	NP	990	neg	neg	neg	neg	pos	Neg	neg	D	CA
	45	M	No	NP	11580	neg	neg	neg	neg	neg	Diazepam<br>Nordiazepam	neg	D	CA
	57	M	No	NP	920	neg	neg	neg	pos	pos	neg	neg	D	CA
	45	M	No	NP	300	neg	neg	neg	neg	neg	Diazepam<br>Nordiazepam	Doxepin	D	CA
	38	F	No	NP	300	neg	neg	neg	pos	pos	neg	neg	D	CA
	27	M	Yes	NP	60	neg	neg	neg	neg	neg	Diazepam<br>Nordiazepam	Ethanol	D	CA
	36	M	Yes	NP	140	neg	neg	neg	neg	neg	neg	Ethanol	N, D	CA
	49	M	Yes	NP	60	neg	neg	neg	neg	neg	neg	Ethanol	N, D	CA
	27	M	No	NP	350	neg	neg	neg	neg	neg	Diazepam<br>Nordiazepam	Doxepin<br>Buprenorphin<br>e	D	CA
	52	F	No	NP	470	neg	neg	neg	neg	neg	neg	Ethanol<br>Trimipramine<br>Nortrimipram<br>ine	D	CA
	32	M	No	NP	1080	neg	neg	neg	pos	pos	neg	neg	D	CA

[42]	41	F	No	NP	17000	neg	neg	neg	neg	neg	neg	neg	-	CN
	26	M	Yes	L: bilateral bronchopneumonia with food aspiration	700	neg	neg	neg	neg	neg	neg	neg	N, P	CA
	45	M	No	L: bilateral bronchopneumonia<br>H: dilated cardiomyopathy with necrosis	900	neg	neg	neg	neg	neg	neg	neg	P, C	CA
	21	M	No	L: bilateral bronchopneumonia<br>H: rheumatic heart disease	500	neg	neg	neg	neg	neg	neg	neg	P, C	CA
	33	M	No	H: hypertrophic cardiomyopathy, replaced mitral valve	800	neg	neg	neg	neg	neg	neg	neg	C	CA
	28	M	No	L: bilateral bronchopneumonia<br>H: hypertrophic cardiomyopathy	600	neg	neg	neg	neg	neg	neg	neg	P, C	CA
	44	M	No	L: pneumonia<br>H: hypertrophic cardiomyopathy	700	neg	neg	neg	neg	neg	neg	neg	P, C	CA
	41	F	No	L: pulmonary inflammation<br>H: acute myocardial ischemia	800	neg	neg	neg	neg	neg	neg	neg	P, C	CA
[36]	38	M	No	NP	1380	neg	neg	neg	neg	neg	Diazepam<br>Nordiazepam	neg	D	CA
	30	M	No	NP	500	neg	pos	neg	neg	neg	neg	neg	D	CA
	24	M	No	NP	280	neg	neg	neg	neg	neg	Bromazepam<br>Nordiazepam<br>7aminoflunitrazepam	Doxepin	D	CA
	39	M	No	NP	30	neg	pos	neg	neg	pos	Diazepam<br>Temazepam	neg	D	CA
	41	F	No	NP	100	neg	neg	neg	neg	neg	Nordiazepam	Ethanol<br>Toxepin	D	CA
	36	F	Yes	NP	470	neg	pos	neg	neg	neg	neg	Paracetamol	N, D	CA
	42	M	Yes	NP	4070	neg	neg	neg	neg	neg	neg	neg	N	CA
	22	M	No	NP	330	neg	neg	neg	neg	neg	Diazepam<br>Nordiazepam<br>Oxazepam<br>Temazepam	THC-COOH	D	CA
	29	M	No	NP	410	neg	neg	neg	neg	neg	Diazepam<br>Oxazepam<br>Temazepam	THC-COOH	D	CA
	36	M	No	NP	170	neg	pos	neg	neg	neg	Diazepam<br>Nordiazepam	Ethanol<br>THC-OH	D	CA

												THC-COOH<br>Tramadol<br>Carbamazepi<br>ne		
	34	M	Yes	NP	260	neg	neg	neg	neg	neg	neg	neg	N	CA
	38	M	No	NP	1350	neg	neg	neg	neg	neg	Diazepam<br>Nordiazepam<br>Oxazepam	neg	D	CA
	32	M	No	NP	170	neg	neg	neg	neg	neg	Bromazepam	Ethanol	D	CA
	20	M	No	NP	430	neg	neg	neg	neg	neg	Diazepam<br>Oxazepam<br>Bromazepam	THC-COOH	D	CA
	21	M	No	NP	520	neg	neg	neg	neg	neg	neg	THC-COOH	D	CA
	24	M	No	NP	90	neg	neg	neg	neg	neg	Diazepam<br>Nordiazepam	THC-COOH<br>Amphetamin<br>e	D	CA
	38	M	No	NP	90	neg	neg	neg	neg	neg	neg	Ethanol	D	CA
	19	M	No	NP	170	neg	neg	neg	neg	neg	neg	neg	-	CN
	40	M	No	NP	830	neg	pos	neg	neg	neg	Diazepam<br>Nordiazepam	THC-COOH<br>Methampheta<br>mine	D	CA
	20	F	No	NP	210	neg	neg	neg	neg	neg	7aminoflunitrazepam	Doxepin	D	CA
	31	M	No	NP	170	neg	pos	neg	neg	neg	neg	Ethanol	D	CA
	35	M	No	NP	160	neg	pos	neg	neg	neg	neg	Ethanol	D	CA
	20	M	No	NP	40	neg	neg	neg	neg	neg	Diazepam<br>Nordiazepam<br>Oxazepam	neg	D	CA
	43	M	No	NP	680	neg	neg	neg	neg	neg	Diazepam<br>Nordiazepam	THC-COOH	D	CA
	32	M	No	NP	310	neg	neg	neg	neg	neg	Diazepam<br>Nordiazepam	Ethanol<br>THC-COOH	D	CA
	18	M	No	NP	70	neg	neg	neg	neg	neg	neg	THC<br>THC-OH<br>THC-COOH<br>Amphetamin<br>e<br>MDMA	D	CA

	28	F	No	NP	540	neg	neg	neg	neg	neg	Diazepam<br>Nordiazepam	neg	D	CA
	28	M	Yes	NP	280	neg	neg	neg	pos	pos	Diazepam<br>Nordiazepam<br>Oxazepam	neg	N, D	CA
	22	F	Yes	NP	390	neg	neg	neg	neg	neg	Diazepam<br>Nordiazepam	THC-COOH	N, D	CA
	44	M	No	NP	1090	neg	neg	neg	neg	neg	neg	neg	-	CN
	44	M	No	NP	2440	neg	pos	neg	neg	neg	neg	neg	D	CA
	30	M	No	NP	180	neg	neg	neg	neg	neg	neg	neg	-	CN
	26	M	No	NP	440	neg	neg	neg	neg	neg	neg	Ethanol	D	CA
	42	M	No	NP	670	neg	neg	neg	neg	neg	neg	THC<br>THC-OH<br>THC-COOH	D	CA
	22	M	No	NP	30	neg	neg	neg	neg	neg	Diazepam<br>Nordiazepam	neg	D	CA
	33	M	No	NP	270	neg	pos	neg	neg	neg	Diazepam	Doxepin	D	CA
	47	M	Yes	NP	830	neg	pos	neg	neg	neg	Diazepam	neg	N, D	CA
	20	M	No	NP	200	neg	neg	neg	neg	pos	neg	neg	D	CA
[96]	37	F	No	L: edema, congestion	1400	neg	pos	pos	neg	neg	Temazepam<br>Diazepam<br>Nordiazepam	Paracetamol	D	CA
[98]	25	M	Yes	L: edema, calcified granuloma<br>H: hypertrophy	450	neg	neg	neg	neg	neg	neg	THC<br>Sertraline<br>Norsertraline	N, C, D	CA
[101]	45	M	No	NP	230	neg	neg	neg	neg	neg	neg	Sertraline	D	CA
[106]	39	M	No	NP	510	neg	neg	neg	neg	neg	Temazepam	Amitriptyline<br>Nortriptyline	D	CA

Table S3. In house cases, with extracted data and classification.

N: number of the case.

Gender. F: female, M: male.

Post-mortem examination. H: heart; L: lung.

neg: no drug detected in blood; np: data not provided.

6-MAM: 6-monoacetylmorphine; BEG: benzoylecgonine; BZDs: benzodiazepine; EDDP: 2-Ethylidene-1,5-dimethyl-3,3-diphenylpyrrolidine.

Other drugs. THC: tetrahydrocannabinol; THC-COOH: tetrahydrocannabinol carboxylic acid.

D: drugs; N: naïve/non-tolerant; P: pulmonary; C: cardiac.

CN: controls. CA: cases

N.	Year	Age	Gender	Naive/non tolerant	Post-mortem examination	Methadone (ng/mL)	6-MAM	Morphine	Codeine	Cocaine	BEG	EDDP	BZDs	Other drugs	Disease or condition	Group
1	2007	32	M	No	Past flebitis, fresh injection marks<br>L: 1560 g, pulmonary edema<br>H: 420g	48	neg	neg	neg	neg	pos	pos	neg	Ethanol	D	CA
2	2008	42	M	No	Fresh injection marks<br>L: edema, mild emphysema<br>H: 550g but with no abnormality	1040	neg	neg	neg	neg	neg	neg	neg	neg	-	CN
3	2009	52	M	No	Fresh injection marks<br>L: 1490g, bilateral edema<br>H: 420g	1400	neg	neg	neg	neg	neg	pos	neg	neg	-	CN
4	2009	41	M	No	Fresh injection marks<br>L: 1820g, hemorrhagic edema, congestion, anthracosis<br>H: 460g, focal areas of sclerosis with normal myocardial cells	550	neg	neg	neg	pos	pos	pos	neg	Promazine<br>Lidocaine<br>Phenacetin	D	CA
5	2009	31	M	Yes	Fresh injection mark<br>L: 1480g, 730g, hemorrhagic edema<br>H: 450g, mild increase in connective tissue	450	neg	pos	neg	neg	neg	neg	neg	Ethanol 0.12	N, D	CA
6	2010	49	M	No	Past injection marks<br>H: 420g<br>L: 1760g, hemorrhagic edema	791	neg	pos	neg	neg	neg	pos	neg	neg	D	CA
7	2010	34	M	No	Past and fresh multiple injection marks<br>L: 1530g, hemorrhagic edema, peribronchial inflammation<br>H: 500g, no abnormality	433	neg	pos	neg	pos	pos	pos	neg	Phenacetine<br>Paracetamol<br>Levamisole<br>Venlafaxine	D, P	CA
8	2010	40	M	Yes	Fresh injection mark<br>L: 1410g, hemorrhagic edema, chronic inflammation	32	neg	pos	neg	neg	neg	neg	neg	Paracetamol<br>Theophylline	N, D, P, C	CA

					H: 360g, edema, myocardial sclerosis and inflammation											
9	2010	37	M	No	Multiple past injection mark<br>L: 1400g, hemorrhagic edema, congestion, chronic inflammation<br>H: 340g, wavy myocardial fibers, edema	338	neg	neg	neg	pos	pos	pos	neg	Ecgonine methyl ester	D, P, C	CA
10	2010	35	M	No	Fresh injection mark. Past pneumonia.<br>L: 1690g, hemorrhagic edema, diffuse lymphomonocytic inflammation<br>H: 460g, edema, myocardial sclerosis, adipose tissue at the right ventricle	1250	neg	neg	neg	neg	neg	pos	neg	Ethanol 0.89<br>Valproate<br>Paracetamol	D, P, C	CA
11	2010	26	M	No	L: 1600g, edema, bronchopneumonia<br>H: 350g	455	neg	neg	neg	neg	neg	pos	neg	neg	P	CA
12	2011	41	M	No	Fresh injection marks<br>L: 940g hemorrhagic dissociation and emphysema<br>H: 320g	495	neg	neg	neg	pos	pos	pos	neg	Ecgonine methyl ester	D, P	CA
13	2012	27	M	No	Multiple past injection marks<br>L: 1505g, hemorrhagic edema<br>H: 300 g, edema	270	pos	pos	pos	neg	neg	pos	neg	neg	D	CA
14	2013	53	F	No	Multiple past injection marks<br>L: 1270g, hemorrhagic edema<br>H: 240g mild coronarosclerosis	5200	neg	neg	neg	neg	neg	pos	neg	Ethanol 2.4	D	CA
15	2013	32	M	No	Fresh injection marks<br>L: 1900g, 880g, hemorrhagic edema, emphysema, bilateral bronchopneumonia<br>H: 440g, severe myocardial sclerosis involving both ventricles, myocardial disarray	209	neg	neg	neg	pos	pos	pos	neg	Ecgonine methyl ester<br>Levamisole<br>Methylecgonine Cinnamate	D, P, C	CA
16	2014	25	F	No	L: 1400g, edema, congestion<br>H: 246g	3350	neg	neg	neg	neg	neg	pos	neg	neg	-	CN
17	2014	46	M	No	L: 1680g, hemorrhagic edema, bronchopneumonia<br>H: 400g, edema	517	neg	neg	neg	pos	pos	pos	neg	Ethanol 0.49<br>Ecgonine methyl ester<br>Levamisole	D, P	CA
18	2014	44	M	No	Nasal mucosa atrophy<br>L: 1980g, hemorrhagic edema, bilateral bronchopneumonia<br>H: 390g, right ventricle myocardial sclerosis, disarray with wavy fibers in the left ventricle	122	neg	neg	neg	pos	pos	pos	neg	Ethanol 0.1<br>Ecgonine methyl ester<br>Methotrimeprazine	D, P, C	CA
19	2014	26	M	No	L: 2010g, 910g, hemorrhagic edema, bronchopneumonia<br>H: 485g myocardial necrosis with myocarditis and pericarditis with lymphogranulocitic inflammation	158	neg	neg	neg	pos	pos	pos	neg	Ethanol 0.1	D, P, C	CA
20	2014	26	F	Yes	L: 1300g, hemorrhagic edema<br>H: 265g, wavy myofibers, sclerosis of papillary muscles	25	neg	neg	neg	pos	pos	pos	neg	Ethanol 0.1,<br>Paracetamol	N, D, C	CA
21	2014	43	F	No	Fresh injection mark<br>L: 1240g, hemorrhagic edema<br>H: 450g	3990	neg	pos	pos	neg	neg	neg	neg	Ethanol 2.35	D	CA

22	2015	44	M	No	Multiple past injection marks and phlebitis<br>L: 1700g, hemorrhagic edema<br>H: 480g. sclerosis myocardial disarray at the septum, wavy myofibers	1450	neg	pos	pos	pos	pos	pos	neg	Ethanol 0.1,<br>Ecgonine methyl ester<br>Fluconazole<br>Levamisole<br>Trimethoprim	D, C	CA
23	2016	38	M	No	Past injection marks and skin necrosis<br>L: 1240g, hemorrhagic edema<br>H: 315g focal severe sclerosis	454	neg	neg	neg	neg	pos	pos	neg	Ethanol 0.1	D, C	CA
24	2016	42	M	No	Past and fresh injection marks<br>L: hemorrhagic edema<br>H: perivasal sclerosis	460	neg	neg	neg	pos	pos	neg	neg	Ethanol 0.1	D, C	CA
25	2016	47	M	No	L: 1650g, edema, bilateral bronchopneumonia<br>H: 400g, wavy myofibers, neutrophils inflammation, lymphomonocytic miopericarditis of both ventricles, sclerosis	187	neg	neg	neg	neg	neg	pos	Delorazepam<br>Lorazepam	Clorpromazine<br>Clotiapine<br>Clozapine	D, P, C	CA
26	2016	19	M	Yes	Fresh injection marks<br>L: 2060g, hemorrhagic edema, endoalveolar inflammation<br>H: 400g, interstitial edema	77	neg	neg	neg	neg	neg	pos	neg	neg	N, P	CA
27	2017	18	M	No	L: 1260g<br>H: 380g	3190	neg	neg	neg	neg	neg	neg	neg	neg	-	CN
28	2018	48	F	No	L: 1780g, hemorrhagic edema<br>H: 320g	2392	neg	neg	neg	neg	pos	pos	neg	neg	D	CA
29	2018	19	F	Yes	L: 640g, hemorrhagic edema, multiple lymphocytic foci<br>H: 210g	82	neg	neg	neg	neg	neg	pos	Alprazolam	Ethanol 0.1	N, D, P	CA
30	2018	43	M	No	L: 1250g, severe hemorrhagic edema, mild emphysema<br>H: 540g, confluential myocardial sclerosis	716	neg	neg	neg	neg	neg	pos	Alprazolam<br>Diazepam<br>Lorazepam<br>Nordiazepam<br>Oxazepam	Ethanol 0.1<br>Mirtazapine	C, D	CA
31	2019	17	M	Yes	L: 1606g. hemorrhagic edema, bronchopneumonia<br>H: 324g, septic emboli, wavy myofibers at septum and right ventricle	122	neg	neg	neg	pos	pos	pos	neg	neg	N, D, P, C	CA
32	2019	47	M	No	L: 2000g, 950g, edema, peribronchial inflammation, emphysema<br>H: 420g	117	neg	neg	neg	neg	pos	pos	neg	THC-COOH	D, P	CA
33	2019	26	F	Yes	L: 960g, hemorrhagic edema<br>H: 310g	1209	neg	neg	neg	pos	pos	pos	neg	Cocaethylene<br>Ephedrine	N, D	CA
34	2020	28	F	Yes	L: 1080g, congestion, edema<br>H: 290g, mild myocardial suffering, edema	645	neg	neg	neg	pos	pos	pos	neg	neg	N, D	CA
35	2020	44	F	No	L: 1020g, multiple foci of bronchopneumonia,hemorrhagic edema, anthracosis, emphysema<br>H: 253g, advanced sclerosis	451	neg	neg	neg	neg	neg	pos	Lorazepam	neg	D, P, C	CA
